# Public Health Nurse Preceptors in Ireland: Exploring Recognition, Preparedness, and Support for Professional Development

**DOI:** 10.1111/phn.70177

**Published:** 2026-08-27

**Authors:** Niamh Vickers, Denise Mc Guinness, Karl F. Conyard, Keighleigh Byrne, Deirdre McEldowney, Jacqueline Austin, Cathy Geraghty, Gonne C. Barry, Kate Frazer

**Affiliations:** ^1^ School of Nursing, Midwifery and Health Systems, Health Sciences Centre University College Dublin Dublin Ireland; ^2^ School of Public Health, Physiotherapy and Sports Science University College Dublin Dublin Ireland; ^3^ School of Medicine, The Royal College of Surgeons in Ireland University of Medicine and Health Sciences Dublin Ireland; ^4^ Health Service Executive Dublin Ireland

**Keywords:** clinical education, preceptorship, preparedness, public health nurses, recognition, supported

## Abstract

**Background:**

Preceptorship is a widely recognized pedagogy which positions experienced nurses as mentors, educators and assessors in the clinical environment. Public Health Nurse (PHN) preceptors are instrumental to the education and development of PHN students. This study aimed to evaluate PHN preceptors’ perceived recognition, preparedness and support in their roles and to determine overall satisfaction.

**Methods:**

A cross‐sectional survey included a validated instrument modified for the Irish community context and convenience sampling methods to target PHN preceptors associated with one Higher Education Institution, following low‐risk ethical approval in 2024. Subscale domains included preparedness, recognition, support and satisfaction. Data were analyzed using descriptive and inferential statistics and STROBE guidelines used for reporting.

**Results:**

A response rate of 35.8% (*n* = 86) was attained. The Preparedness domain demonstrated the highest consensus (*M* = 12.48, SD = 2.06), indicating that respondents felt prepared for their Preceptorship role. Respondents demonstrated high levels of agreement with pedagogical competencies related to making objective student assessments and supporting skills development. Barriers included insufficient time to engage with preceptorship. The results reveal a disconnect between preceptors perceiving that they were highly valued by students but that the preceptorship role was not formally acknowledged as part of their workload at an organizational level. High attendance was reported at preceptor education sessions (87.2%) and a preference for future education to be delivered online (46.4%).

**Conclusion:**

PHN preceptors are critical to integrating theoretical learning in the clinical practice environment. While results demonstrate high satisfaction and confidence, significant challenges exist at the systems level to balance the role and demands of preceptorship, within workloads due to time constraints. At a time when primary care settings are being prioritized for healthcare, the workforce challenges need to be understood and solutions co‐created. Future research should incorporate qualitative methods to explore the experiences of preceptors and PHN students.

**Clinical Trial Registration:**

Clinical trial registration was not applicable to the study.

## 1 Introduction

Within the Republic of Ireland (ROI), PHNs provides an overarching, integrated service encompassing health promotion, education, and disease prevention for individuals, families, and communities. Adopting a population‐health perspective, PHNs are responsible for identifying and addressing the wider social determinants of health, fundamentally influencing community well‐being (NMBI 2023; McDonald et al. 2013; McDonald et al. 2015). As pivotal professionals in the delivery of essential community healthcare services, PHNs in Ireland manage nursing care across the entire lifespan, ranging from maternal and infant health to the care of the older adult (McDonald et al. 2013; McDonald et al. 2015). High quality academic and clinical education are the cornerstone in the preparation of future generations of PHNs to become competent practitioners. PHN preceptors are educational facilitators who demonstrate their professional competence as practitioners supporting student nurses in clinical practice settings (Hardie et al. 2025). Despite long‐standing recognition of nurse preceptors being critical in the undergraduate student's journey (Hardie et al. 2025), there is limited understanding of how PHN preceptors are recognised, prepared and supported within the Irish healthcare landscape in their role supporting student public health nursing during their graduate registration program.

Preceptorship is a widely recognized pedagogy used to educate nursing students in clinical settings worldwide. Preceptors provide education, mentorship and act as assessors for students in clinical practice. They have a fundamental role in validating students’ knowledge, skills and understanding while also acting as role models demonstrating practice in a competent manner (Ulrich et al. 2025). Preceptors are instrumental in PHN education and their role is invaluable given that more than half (60%) of the academic program is located in the clinical practice setting. The Nursing and Midwifery Board of Ireland (NMBI), requires students completing the Graduate Diploma in Public Health Nursing to complete a minimum of six‐hundred and seventy‐five hours in clinical practice environments of the Public Health Nursing Service (NMBI 2023). The duration of the Graduate Diploma in Public Health Nursing is one academic year running from September to June and thus the clinical practicum in the PHN service is a vital component of the program and underpins the imperative role of the PHN preceptor in the clinical environment. During this time the student PHN (preceptee) and the PHN (preceptor) work closely together, developing the students’ knowledge and skills development in the application of theoretical learning to Public Health Nursing practice and supporting the achievement of registration standards, requirements learning goals. PHN preceptors objectively assess the competence and skills of student PHNs in the practice environment and thus play an imperative role.

Unfortunately, the PHN service faces numerous workforce challenges in the current climate with staff shortages being of particular concern. Recent data highlighted a marginal 1% increase in PHN's practicing nationally (NMBI 2025). While this is a positive step, recruitment and retention of PHNs remains an issue in highly populated cities including Dublin, where staff recruitment and retention are significant issues. The shortcomings in the PHN service are acknowledged by policymakers, including delays in developmental screening of children due to workforce challenges and staffing noted by the Irish Government (Houses of the Oireachtas 2024). In 2024 staffing issues were further highlighted by the rising post‐Covid‐19 workplace turnover, which reached 7.4% (HSE 2024). This instability in the healthcare sector has directly affected the number of experienced nurses available to preceptor students. These challenges are not new, as PHNs reported their concerns with the working environment providing and measuring care practices. Giltenane et al. (2022) noted absence of appropriate supports and resources means PHNs work hard to provide quality care.

In addition to the structural challenges faced by PHN preceptors’ nationally, the international literature suggests that there are several factors which inhibit nurses specifically in their roles as preceptors. Barriers include a lack of formal education to prepare them for their preceptorship role and a lack of organizational supports (Hardie et al. 2025; Ibrahim et al. 2024). Recent evidence further confirms that it is common practice for preceptors to be delegated a preceptorship role without any formal education or training (Hardie et al. 2025, Chan et al., 2025, Regaira‐Martínez et al. 2024). Preceptors report that their role as preceptor is not recognized by the organization and they report feeling unsupported They described feeling ‘burned out’ and overwhelmed in balancing their clinical responsibilities and duties with the supporting the educational development of nursing students.

Giltenane et al. (2022) and McDonald et al. 2013; McDonald et al. 2022) confirm the challenges of the work environment for PHNs, including home visiting, workload demands, and lack of resources. In addition, Preceptor PHNs have an additional responsibility to provide quality education to students meeting the NMBI standards and requirements; however, they do not receive formal recognition or incentives. The workload is expected to be absorbed on top of their caseload responsibilities and remains ‘invisible’ (Heggarty and Foran 2025, Ibrahim et al. 2024; McDonald et al. 2013; McDonald et al. 2015). The lack of recognition and organisational supports such as protected teaching time from clinical workload has been found to impede their ability to educate students (Davis 2024).

Lack of institutional support along with limited educational and career development opportunities also leads to a lack of incentive and an increased level of burnout for preceptors (Ibrahim et al. 2024). In contrast, in areas where preceptors are supported by their organisation receiving formal education, including competency‐based training and formal recognition, leads to lower attrition rates. Preceptors who participate in evidence‐based continuous professional development report higher competence and confidence in their role as preceptors, leaving them in a stronger position to provide quality education to students (Ulrich et al. 2025).

From an Irish perspective, there is limited research exploring the PHN preceptors’ experiences. There is a significant lack of insight into PHNs’ experiences of career development, organisational supports and training, leading to limited data on their overall preparedness to fulfil their role as preceptors. There are no minimum standards required to be appointed as a PHN preceptor in practice and there is no formal academic education program. While Giltenane et al. (2022) provided an insight into workload and resource challenges for Irish PHNs, we have no understanding of the specific preceptorship role or demands. This study addresses a critical gap in empirical research by exploring recognition, preparedness and support for professional development of PHNs who are formal clinical preceptors and supporting graduate student PHNs.

## 2 Methodology

### 2.1 Aim/ Objectives

The aim of this study was to evaluate PHN preceptors perceived recognition, preparedness and support in their role as a PHN preceptor. The objectives of this study were to: (1) ascertain if PHN preceptors feel recognized for their role as a PHN preceptor (2) explore if PHN preceptors feel prepared for their role as a PHN preceptor (3) investigate if PHN preceptors feel supported in their role as a PHN preceptor (4) evaluate satisfaction with the PHN preceptor role.

### 2.2 Design and Setting

This study utilized an online cross‐sectional survey design to collect data regarding PHN preceptorship practices across Ireland.

### 2.3 Ethical Approval

Low risk ethical approval to conduct the study was granted by University College Dublin Human Research Ethics Committee (LS‐LR‐24‐262‐Vickers) on 26^th^ July 2024.

### 2.4 Sample and Recruitment

To be included in the study respondents must have been a preceptor to a PHN student in one specific University in Ireland over the previous two years. The University is one of three Higher Education Institutions (HEIs) in Ireland that offers the Graduate Diploma in Public Health Nursing. This HEI has the largest intake of PHN students nationally. Respondents were recruited using convenience sampling through a targeted email which was circulated to all PHNs via an Assistant Director of Public Health Nursing who was acting as liaison. The email included an information flyer, participant information leaflet and a link to the survey. Secondly, we accessed two Facebook pages: one specifically for PHNs in Ireland and Nursing/ Midwifery in Ireland and shared information about the study and a link to the survey.

### 2.5 Measurement Instrument

The measurement instrument utilized in this study is a previously validated tool, specifically developed in Australia by Cusack et al. (2020), to evaluate registered nurses (RNs) teaching and supervision of undergraduate nursing students in acute clinical environments. Permission to use the instrument was provided by the lead author to utilize, modify and contextualize the instrument for use within the Irish context, specifically within the community healthcare setting (Cusack et al. 2020). To ensure content validity and clinical relevance, the lead author and another member of the research team, an Assistant Director of Public Health Nursing Practice Development Coordinator (ADPHN PDC) performed primary modifications to the instrument. Subsequently, this was subjected to a phase of expert panel synthesis to ensure the instrument was relevant and appropriate for PHNs (Koller et al. 2017). The research team comprised Directors of Public Health Nursing (DPHN), ADPHN PDC, and academics who provided feedback and suggested contextual modifications ensuring that the nuances of the PHN preceptor role were assured while retaining the original domains: recognition, preparedness and support. A pilot study was conducted with four experienced PHNs, academics and ADPHN PDC. This included usability testing of the online survey via the Qualtrics platform. Feedback was provided, and minor adaptations were completed to ensure the instrument was relevant. The final instrument consisted of 26 items across five distinct sections: demographics and workload, a four‐point Likert scale for evaluation of the domains of recognition, preparedness and support and a final evaluative section utilizing binary, categorical and satisfaction scales to measure satisfaction, the challenge of balancing preceptorship and PHN practice and future educational preferences for preceptorship training.

### 2.6 Data Collection and Analysis

The survey used an online Qualtrics platform for anonymous data collection. To ensure respondent anonymity, no identifying details were collected in the survey such as names or email addresses. Further, the anonymize responses feature was enabled in Qualtrics to ensure that no IP addresses or location data were recorded from the respondents. Data were exported from Qualtrics to the IBM Statistical Package for the Social Sciences (SPSS) and analyzed using descriptive and inferential statistics. A one‐way analysis of variance (ANOVA) was conducted to compare the overall domain mean scores of highest education status to determine if significant differences existed between groups based on highest educational attainment. The threshold for statistical significance was *p* < 0.05. Statistical tests met the assumption of homogeneity of variance using Levene's test, and the assumption of normality using the Kolmogorov‐Smirnov tests.

## 3 Results

### 3.1 Respondent Characteristics

A response rate of 35.8% was achieved (*n* = 86/240), and representing eight of the nine community health areas nationally (Table 1). Public Health Nurses (PHNs) were Registered General Nurses and Registered Public Health Nurses. Several participants held additional professional registrations as detailed in Table 1, including Registered Midwife (*n* = 32, 37.2%) and Registered Children's Nurse (*n* = 17, 19.8%). More than a quarter of respondents (*n* = 22, 26.5%) had obtained a Master's degree. The average length of time since qualifying as a PHN was 11.38 years. Notably, the average time since qualifying as a PHN and assuming the role of a preceptor PHN was 6.35 years. In relation to caseload profile, the average caseload representing child health was 234.55 children and clinical nursing caseload was 81.2 individuals (Table 2).

**TABLE 1:** Respondent characteristics.

Variable	n	(%)
**Professional Registration Type** ^*^		
Registered Public Health Nurse	86	(100)
Registered General Nurse	86	(100)
Registered Midwife	32	(37.2)
Registered Children's Nurse	17	(19.8)
Registered Mental Health Nurse	1	(1.2)
Registered Intellectual Disability Nurse	0	(0)
Registered Nurse Tutor	1	(1.2)
Other	3	(3.5)

Highest Level of Education		
Graduate Diploma	61	(73.5)
Master's Degree	22	(26.5)

What CHO^**^ are you working in?		
CHO 1	9	(26.6)
CHO 2	2	(2.4)
CHO 3	4	(4.8)
CHO 5	7	(8.3)
CHO 6	13	(15.5)
CHO 7	17	(20.2)
CHO 8	15	(17.9)
CHO 9	17	(20.2)

* **Professional Registration Type**: a practitioner can hold multiple registrations.

** **CHO**: Community Healthcare Organisation.

**TABLE 2:** Professional history and case‐mix of respondents.

Variable	*n*	Range min‐max	Mean	SD
How many years are you qualified as a nurse?	54	(8–39)	21.78	7.187
How many is in your Child Health Caseload?	73	(21–550)	234.55	107.575
How many is on your Clinical Case Load?	74	(0–600)	81.2	85.854
How many years are you qualified as a PHN^*^?	84	(2–37)	11.385	7.2343
How many years were you qualified when you were a	81	(1–22)	6.351	4.678
PHN preceptor to a student undertaking the Graduate				
Diploma in Public Health Nursing?				

* **PHN**: Public Health Nurse.

### 3.2 Perceptions of Preparedness, Recognition and Support

The respondents’ perceptions of their PHN preceptorship experience were analyzed across three primary domains: preparedness, recognition and support. Aggregate scores were calculated for the three primary domains (Table 3). As the survey utilized a four‐point Likert scale ranging from 1 (strongly disagree) to 4 (strongly agree). A one‐way analysis of variance ANOVA revealed that higher educational attainment significantly influenced the overall mean scores for the preparedness domain (*F* = 1.90, *p* = 0.022) but had no significant influence on the total recognition (*F* = 1.42, *p* = 0.131) or support domains (*F* = 1.39, *p* = 0.148) as detailed in Table 4. However, item level analysis showed that education significantly influenced PHN preceptor perceptions of student recognition (*F* = 2.28, *p* = 0.005), feeling valued by students (*F* = 2.05, *p* = 0.012), and support received from the University (*F* = 1.84, *p* = 0.027) (Table 4).

**TABLE 3:** Overall scores on recognition, preparedness and support domains.

Domain^*^	n	Minimum score	Maximum score	Mean score	Standard deviation
Recognition	84	15	24	19.58	2.40586
Preparedness	84	8	16	12.48	2.06808
Support	86	4	16	10.53	2.46719

* Domain of recognition contained six questions, while the preparedness and support each contained four questions. All items within these domains were scored on a four point Likert scale (where 1 = strongly disagree, and 4 = strongly agree).

**TABLE 4:** One way analysis of variance (ANOVA) for PHN preceptors perceptions of recognition, preparedness and support by highest educational attainment.

Question	SS	df	Mean Square	F	*p*‐value
**Recognition domain**					
Total recognition score	197.61	27	7.31	1.42	**ns**
I feel that my direct line manager recognizes my contribution to the teaching and supervision of PHN students	10.35	27	0.38	1.17	**ns**
I feel that the teaching and supervising of PHN students is acknowledged by my line manager as part of my workload	13.66	27	0.50	1.08	**ns**
I believe that the teaching and supervision of PHN students is a part of my role as a RPHN	9.41	27	0.35	1.17	**ns**
I think that PHN students recognize my role in their teaching and supervision	11.82	27	0.44	2.28	**.005** ^**^
I feel valued by PHN students when providing teaching and supervision	13.82	27	0.51	2.05	**.012** ^*^
I feel that my line manager(s) recognize my role in the teaching and supervision of PHN students	10.35	27	0.38	1.17	**ns**
**Preparedness Domain**					
Total preparedness score	172.71	27	6.40	1.90	**.022** ^*^
I am equipped to balance my caseload to prioritize independent learning by PHN students	17.17	27	0.64	1.16	**ns**
I feel prepared to teach and supervise PHN students in the practice environment	17.04	27	0.63	2.14	**.008** ^**^
I feel prepared to support PHN students in developing their skills and applying their knowledge	13.43	27	0.50	2.16	**.008** ^**^
I feel confident in my ability to make objective, unbiased assessment decisions regarding the progress of PHN students	8.40	27	0.31	1.10	**ns**
**Support Domain**					
Total support score	205.86	27	7.62	1.39	**ns**
I feel I have enough time to engage in teaching and supervision of PHN students within the practice environment	26.65	27	0.99	1.53	**ns**
I feel there are adequate supports for PHNs taking on teaching and supervision roles of PHN students	20.34	27	0.75	1.15	**ns**
I feel that the University supports my role in teaching and supervising of PHN students	15.14	27	0.56	1.85	**.027** ^*^
I feel supported by line management in my teaching and supervision of PHN students	17.90	27	0.66	1.36	**ns**

* Statistically Significance;

** Moderate Statistical Significance; **ns** = Not Significant.

### 3.3 Preparedness

The preparedness domain (4 items) demonstrated the strongest level of consensus among the cohort (*M* = 12.48, *SD* = 2.06) indicating that respondents felt prepared for their Preceptorship role. A detailed analysis of the individual four items within the domain demonstrated a clear distinction between the preceptor's confidence in their teaching abilities and their capacity to manage the logistical demands of the role, as detailed in Table 5. Preceptors reported the strongest consensus in their ability to make objective assessment decisions (*M =* 3.39, *SD =* 0.53), their readiness to support in their skill development and application of knowledge (*M =* 3.21, *SD =* 0.55) and in their preparedness to teach and supervise PHN students in the clinical environment (*M =* 3.13, *SD =* 0.62). In contrast, the item relating to the ability to balance their caseload and prioritise independent learning by student PHNs (*M =* 2.76, *SD =* 0.75), represented the lowest level of agreement in the preparedness domain.

**TABLE 5:** Individual question scores on recognition, preparedness and support domains.

Question	Mean	SD
**Recognition Domain**		
I feel that my direct line manager recognizes my contribution to the teaching and supervision of PHN students	3.21	0.63
I feel that the teaching and supervising of PHN students is acknowledged by my line manager as part of my workload	3.11	0.69
I believe that the teaching and supervision of PHN students is a part of my role as a RPHN	3.40	0.55
I think that PHN students recognize my role in their teaching and supervision	3.44	0.52
I feel valued by PHN students when providing teaching and supervision	3.37	0.57
I feel that my line manager(s) recognize my role in the teaching and supervision of PHN students	3.06	0.58
**Preparedness Domain**		
I am equipped to balance my caseload to prioritise independent learning by PHN students	2.76	0.75
I feel prepared to teach and supervise PHN students in the practice environment	3.13	0.62
I feel prepared to support PHN students in developing their skills and applying their knowledge	3.21	0.55
I feel confident in my ability to make objective, unbiased assessment decisions regarding the progress of PHN students	3.39	0.53
**Support Domain**		
I feel I have enough time to engage in teaching and supervision of PHN students within the practice environment	2.45	0.86
I feel there are adequate supports for PHNs taking on teaching and supervision roles of PHN students	2.52	0.82
I feel that the University supports my role in teaching and supervising of PHN students	2.79	0.61
I feel supported by line management in my teaching and supervision of PHN students	2.77	0.73

### 3.4 Recognition

The recognition domain (6 items) yielded a mean score (*M =* 19.58, *SD =* 2.40) which reflects a high level of agreement among respondents. However, while the overall domain indicated a positive agreement, item‐level analysis revealed that recognition is most strongly felt through student interaction and professional identity as a PHN rather than managerial acknowledgment and recognition. Notably, the highest level of agreement related to student interactions with respondents feeling that students recognized their role (*M =* 3.44, *SD =* 0.52) and feeling valued by students when they provided teaching and supervision (*M =* 3.37, *SD =* 0.57). There was a strong consensus that teaching and supervising PHN students was part of the PHN role (*M =* 3.40, *SD =* 0.55). Contrastingly, scores were notably lower regarding managerial recognition, indicating less agreement compared to recognition from students. Respondents felt less certain that their contribution was acknowledged as part of their formal workload by line managers (*M =* 3.11, *SD =* 0.69) or that their role in teaching and supervision of PHN students was recognized by managers (*M =* 3.06, *SD =* 0.58).

### 3.5 Support

The support domain (4 items) returned a mean score of (*M =* 10.53, *SD =* 2.46) indicating the area of highest dissatisfaction among participants. Item‐level analysis identified this domain as the area of highest relative dissatisfaction and the most significant perceived barrier within the preceptorship role. The item in relation to time allocation to engage in teaching and supervision of PHN students within the practice environment returned the lowest mean score across the entire survey (*M =* 2.45, *SD =* 0.86), indicating a consensus of disagreement. In relation to overall supports for the preceptorship role, respondents indicated only a marginal level of agreement with the item (*M =* 2.52, *SD =* 0.82). Of note, while the overall domain score was not significantly influenced by education level it was noted that educational attainment did significantly influence perceptions of support received from the University (*F* = 1.84, *p* = 0.027). Participants reported comparable levels of agreement regarding support from the University (*M =* 2.79, *SD =* 0.61) and from direct line management (*M =* 2.77, *SD =* 0.73).

### 3.6 Satisfaction and Evaluation

The final section of the survey evaluated overall satisfaction levels, ascertaining if balancing preceptorship and their caseload was challenging. Finally, respondents were asked to indicate if they had attended the preceptor information sessions and what mode of education they would choose for future education in preceptorship. Respondents reported high levels of satisfaction with the role and 95.3% (*n* = 82) reported that they were satisfied with the education sessions provided. The majority of respondents (87.2%, *n* = 75) reported that they attended the preceptor information sessions and the majority stated their future preferences would be synchronous education sessions delivered online (46.4%, *n* = 39), followed by in‐person sessions (34.5%, *n* = 29) and asynchronous sessions with a question and answer option (19%, *n* = 16).

## 4 Discussion

This study was undertaken to address the absence of empirical research specifically on PHN preceptorship within the Irish context. By providing the first dedicated investigation of the perceived preparedness, recognition and support of PHN preceptors within the geographical context of Ireland. This research offers a foundational contribution to the field and provides important insights to PHN preceptor experiences.

Notably, the study revealed a remarkably high level of satisfaction among respondents with almost all PHN preceptors (95.3%, *n* = 82) reporting a positive experience with the PHN preceptor role. This finding is consistent with other empirical investigations which suggest that despite challenges, the role is fundamentally perceived as a rewarding and positive experience (Regaira‐Martínez et al. 2024, Girotto et al. 2019, Bowen et al. 2019). Within our current study, this high level of satisfaction appears to be underpinned by the PHNs perceived professional identity where the majority of respondents agreed that teaching PHN students is an inherent part of the PHN role.

Nevertheless, despite the high levels of satisfaction among respondents, a prominent finding in this study was the significant challenge of balancing the dual role of PHN preceptorship with PHN practice. This was evidenced in multiple data points within the survey. In the categorical variable, the majority (70.9%, *n* = 61) explicitly reported that balancing preceptorship with their caseload was challenging. This finding is further evidenced specifically within the preparedness domain where the lowest level of agreement attained related to the preceptor's ability to balance caseload management and support student PHN learning. While the PHN preceptors were personally prepared and confident in relation to the teaching, supervision, assessing and supporting of PHN students, it is evident that the logistical aspect of preparedness, specifically relating to balancing clinical workload and prioritising student learning is absent which is consistent with current literature (Heggarty et al. 2025, Ibrahim et al. 2024). It has been identified previously that preceptorship can be perceived as challenging and stressful due to an intensified workload which can ultimately precipitate burnout and reduced job satisfaction (Livingstone 2024, Smith et al. 2024, Valizadeh et al. 2016). An interesting finding in our study was that higher educational attainment significantly influenced the respondents’ perceptions of preparedness (*F* = 1.90, *p* = 0.022) which suggests that educational background may contribute to how PHN preceptors perceive their readiness for the pedagogical aspects of the role. The influence of educational background on PHN preceptors has been previously documented by Larsen and Zahner (2011) who ascertained that PHN preceptors with higher academic qualifications consistently reported higher self‐efficacy scores across multiple timepoints.

Within the support domain, it was explicitly noted that PHN preceptors did not have sufficient time to engage in teaching and supervision in their clinical areas. This criterion received the lowest mean score on the survey (*M = 2.45)* representing the highest level of disagreement and indicating time constraints as a prominent issue within the contemporary clinical environment of PHN in Ireland. Indeed, it can be argued that this is underpinned by contextual factors such as high caseloads and short staffing within the national PHN service in Ireland. Time constraints are a well‐established challenge for preceptors for undergraduate students in the literature (Smith et al. 2024, Cusack et al. 2020, Benny et al. 2023, Benny et al. 2024). Notably, it was highlighted in a recent integrative review that organizations should strategically adjust clinical workloads to provide protected time for preceptors to cultivate their pedagogical skills (Heggarty et al. 2025).

This current study highlights a clear distinction between the varying sources of support for PHN preceptors. While respondents felt supported by the University (*M =* 2.79) and their direct line management (*M =* 2.77), there is a notable contrast with the lower level of agreement regarding the adequacy of overall supports (*M =* 2.52). Additionally, PHN preceptors’ level of education significantly influenced their perception of University support (*F* = 1.84, *p* = 0.027). This suggests that a preceptor's academic background and engagement with further professional development may shape their engagement with HEI resources and supports. However, while organizational supports may enhance the preceptorship experience it may not necessarily alleviate or mitigate the inherent barriers and challenges of the role (Kirkpatrick et al. 2025, Christopherson et al. 2025, Ibrahim et al. 2024).

The findings from our current study reveal an explicit disconnect between how PHN preceptors feel recognised individually and at an organisational level. Interestingly, the respondents felt most recognised and valued by the PHN students themselves, with the highest levels of agreement related to these criteria. Preceptors’ educational attainment was found to significantly influence whether they felt recognized (*F* = 2.28, *p* = 0.05) and valued (*F* = 2.05, *p* = 0.012) by PHN students indicating that these are perceived differently based on educational attainment. However, this agreement was reduced overall when respondents were asked if teaching and supervising PHN students are acknowledged by line managers, as part of their formal workload or if managers recognise the role in a general capacity. Our results are consistent with Cusack et al. (2020) and Smith et al. (2024) and confirms the preceptor role is frequently not recognised at a systems level. Our findings demonstrate significant professional engagement with preceptor information sessions provided by the University and high attendance. Further developments for HEIs need to prioritise participation and preference for synchronous online educational sessions.

## 5 Strengths, Limitations And Implications

This study possesses several significant strengths that makes a novel contribution to contemporary PHN preceptorship. Methodologically, the study utilized a previously validated tool. The response rate of 35.8% is low; however, we acknowledge that respondents in the study may not represent the experiences of a majority of PHNs working as Preceptors in practice and are not nationally representative. The sample only included PHN preceptors supporting students attending one HEI in Ireland. While this HEI has the largest intake of PHN students nationally, the findings may not be generalizable to preceptors working within other HEIs. PHN preceptor education is currently provided individually by each HEI. The implementation of the Public Health Nursing National Clinical Assessment Document (NCAD) across the three HEIs that offer the Graduate Diploma in Public Health Nursing came into effect in September 2025. The findings of this study and the move towards standardization across the HEI's is an opportune time for establishing a unified national PHN preceptor educational model in Ireland. Such a model would ensure that all PHN preceptors nationally receive consistent, evidence‐based preparation for the role. Future educational models should support online digital delivery to support access to the national PHN workforce. Future research directions should explore the experiences of PHN preceptors and from student PHNs in Ireland to gain richer and more comprehensive insights. Future studies should consider further data collection and psychometric validation of this adapted instrument in the Irish community context. Additionally, a preceptor educational model should be developed with stakeholder involvement.

## 6 Conclusion

This primary research represents the first study in Ireland specifically investigating the recognition, preparedness and support of PHN preceptors in Ireland, offering important and foundational insights into the vital role of the PHN Preceptor. Our findings highlighted that PHN preceptors are overall satisfied with their role. Nevertheless, a prominent finding is the systemic challenge of balancing preceptorship along with PHN practice. While PHN preceptors are pedagogically prepared and confident in their ability to assess students, the lack of time and formal workload recognition are significant challenges in the contemporary PHN landscape.

## Funding

This research did not receive funding.

## Ethics Approval Statement

Low risk ethical approval was granted from University College Dublin.

## Patient Consent Statement

Patient consent statement was not applicable to the study.

## Conflicts of Interest

The authors have declared that no competing interests exist.

## Permission to Reproduce Material From Other Sources

Permission to reproduce material from other sources was not applicable to the study.

## Supporting information




**Supporting Information 1**: phn70177‐sup‐0001‐SuppMat.docx

## Data Availability

All relevant data are within the manuscript and its supporting information files.
